# Climate of Upper Georgia

**Published:** 1877-02-20

**Authors:** 


Climate of Upper Georgia.—A gentleman in St. Joe, Cal., writes us to learn something of the climate and health of this section of country. We refer him and others desiring such information, to Dr. Little, the State Geologist, Atlanta, Ga., and to the editor of *The South*, New York, who has devoted a copy of his paper to a description, etc., of each of the Southern States. We will add that, excepting about three and a half months, dating from about the middle of November, during which the weather is variable, with occasional rains followed by cold snaps, rarely falling below 30° F., our climate is mild, bracing and healthful. The water, particularly in Middle and North-east Georgia, is superb.

